# The current state of artificial intelligence in endoscopic diagnosis of early esophageal squamous cell carcinoma

**DOI:** 10.3389/fonc.2023.1198941

**Published:** 2023-05-24

**Authors:** Yuwei Pan, Lanying He, Weiqing Chen, Yongtao Yang

**Affiliations:** ^1^ Department of Gastroenterology, Chongqing University Cancer Hospital, Chongqing, China; ^2^ Chongqing Key Laboratory of Translational Research for Cancer Metastasis and Individualized Treatment, Chongqing University Cancer Hospital, Chongqing, China

**Keywords:** artificial intelligence, convolutional neural network, endoscopy, esophageal squamous cell carcinoma, diagnosis

## Abstract

Esophageal squamous cell carcinoma (ESCC) is a common malignant tumor of the digestive tract. The most effective method of reducing the disease burden in areas with a high incidence of esophageal cancer is to prevent the disease from developing into invasive cancer through screening. Endoscopic screening is key for the early diagnosis and treatment of ESCC. However, due to the uneven professional level of endoscopists, there are still many missed cases because of failure to recognize lesions. In recent years, along with remarkable progress in medical imaging and video evaluation technology based on deep machine learning, the development of artificial intelligence (AI) is expected to provide new auxiliary methods of endoscopic diagnosis and the treatment of early ESCC. The convolution neural network (CNN) in the deep learning model extracts the key features of the input image data using continuous convolution layers and then classifies images through full-layer connections. The CNN is widely used in medical image classification, and greatly improves the accuracy of endoscopic image classification. This review focuses on the AI-assisted diagnosis of early ESCC and prediction of early ESCC invasion depth under multiple imaging modalities. The excellent image recognition ability of AI is suitable for the detection and diagnosis of ESCC and can reduce missed diagnoses and help endoscopists better complete endoscopic examinations. However, the selective bias used in the training dataset of the AI system affects its general utility.

## 1 Introduction

Esophageal cancer (EC) is a malignant tumor originating from the esophageal mucosal epithelium and is one of the most common malignant tumors of the digestive tract. The incidence rate and incidence patterns of esophageal cancer vary significantly among different countries and regions. East Asia has the highest incidence rate, which can reach twice that of the world average level (12.2/100,000) (1). The pathological type is mainly esophageal squamous cell carcinoma (ESCC), which accounts for more than 90% of cases in China (2). In relatively low-incidence areas, such as Europe and the United States, the pathological type is mainly adenocarcinoma (3). It was estimated that in 2020 there would be 604,000 new cases of esophageal cancer worldwide (accounting for 3.1% of all cancers), along with its incidence rate ranking tenth among all malignant tumors (the standardized incidence rate is 9.3/100,000 for males and 3.6/100,000 for females), and 544,000 deaths (accounting for 5.5%), and the mortality rate would rank sixth among malignant tumors (the standardized mortality rate is 8.3/100,000 for males and 3.2/100,000 for females) (1). China is an important country for esophageal cancer and according to the latest cancer report released by the National Cancer Center in 2019, 246,000 new cases of esophageal cancer and 188,000 deaths were recorded in China in 2015 (4). The incidence rate and mortality rate ranked sixth and fourth, respectively, among all malignant tumors, accounting for 53.7% and 55.7% of the global total, respectively (5). A total of 70% of patients with esophageal cancer had lost the opportunity for surgery due late detection and a high tumor burden (4).

Most early ESCC and precancerous lesions can be treated using minimally invasive methods of treatment performed under an endoscope, with a 5-year survival rate of patients being as high as 90% (6–8). Patients with advanced ESCC have a low quality of life and a poor prognosis, and the overall 5-year survival rate is less than 20% (9). At present, the early diagnosis rate of esophageal cancer is still low (4). Most patients are diagnosed after developing progressive dysphagia or metastatic symptoms, and the tumor is often in the middle or late stages by this time. The most effective method of reducing the disease burden in areas with a high incidence of esophageal cancer is to prevent the disease from developing into invasive cancer. Due to the lack of typical clinical symptoms during early esophageal cancer, the key to improving the early diagnostic rate of ESCC is to screen high-risk populations. However, because of the uneven professional level of endoscopists, there are still many missed cases due to failure to recognize lesions. Endoscopic screening program in high-risk areas of ESCC also leads to an increased workload of endoscopists. Studies have showed computer-aided endoscopic monitoring can help detect and classify suspicious lesions, thereby improving the detection rate of ESCC (10–13). Artificial intelligence (AI)-assisted endoscopic diagnosis has shown promising prospects to solve the problems of the sharp increase in the workload and low inspection efficiency (14–16). In this review, we summarize the current status of utilizing AI for endoscopic detecting of early ESCC.

## 2 Current endoscopic screening techniques for esophageal squamous cell carcinoma

In regions with a high incidence of esophageal cancer, early detection of esophageal cancer and intraepithelial neoplasia are recommended as the primary objective for screening. The screening guidelines in China suggest an initial age of 45 years for esophageal cancer screening, with screening to be ceased at 75 years old or when life expectancy is less than 5 years. For those who meet the screening age, screening should be focused on the following high-risk groups: 1) individuals born or residing in areas with a high incidence of ESCC; 2) those who have a family history of ESCC; 3) those with known high-risk factors for esophageal cancer, such as smoking, excessive alcohol consumption, squamous cell carcinoma of the head and neck or respiratory tract, preference for high-temperature and pickled foods, and poor oral hygiene (17).

Upper gastrointestinal endoscopy is still the gold standard for the diagnosis of esophageal cancer (17). Along with the popularization of endoscopy and pathological biopsy, the detection and diagnosis rates of early esophageal cancer have increased significantly. Among them, ordinary white light imaging (WLI) endoscopy is a widely used routine examination method, and the early cancer diagnosis rate can reach 80% along with the assistance of a biopsy (18). It is a basic technology used for screening early cancer and is of great significance for the discovery and diagnosis of esophageal cancer.

However, due to limitations of macroscopic morphological judgment by the naked eye, the accuracy of biopsy, and the expertise of examiners, WLI endoscopy can lead to missed diagnoses of precancerous lesions and early esophageal cancer. WLI endoscopy combined with Lugol chromoendoscopy (LCE) is currently the standard method used for screening ESCC and precancerous lesions (17). This method is based on the principle that glycogen in the non-keratinized epithelium turns brown when it encounters iodine. When the esophageal mucosa is diseased, the amount of glycogen decreases, so the color becomes lighter or even disappears, forming a sharp contrast with normal stained mucosa, which is helpful for the identification, positioning, and targeted biopsy of the lesion. This method can improve the detection rate of early esophageal cancer, microcarcinoma, and precancerous lesions with an accuracy rate of 90–100% (19, 20). However, some patients experience discomfort, including heartburn, chest pain, or even severe allergic reactions after iodine staining.

At well-equipped endoscopic centers, upper gastrointestinal WLI endoscopy combined with electronic staining imaging can be used as the preferred screening method. Among them, narrow-band imaging (NBI) improves the sensitivity of early EC diagnosis to more than 90% compared with ordinary WLI endoscopy (19, 21). Electronic staining imaging technology mainly conducts special optical processing on the digestive tract mucosa to more clearly display the fine structure and superficial blood vessels on the mucosal surface. At the same time, there is no adverse reaction to chromoendoscopy dyes, so it is widely used in clinical practice to guide the determination of the range of suspected early esophageal cancer lesions and tissue biopsy. During recent years, the combination of magnifying endoscopy and electronic staining endoscopy has created powerful image enhancement technology that has been widely used in the field of digestive endoscopy. Using a magnifying electronic staining endoscope after ordinary WLI endoscopic observations can first define the range of the lesion and then aid the observation of the morphology of mucosal capillaries in the lesion area. It is an efficient early cancer diagnosis method, and its sensitivity to lesions can reach 95% (21).

**Figure 1:**
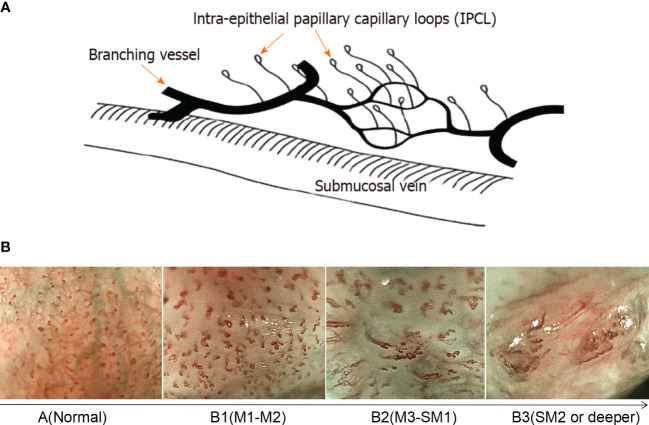
Prediction of the invasion depth of early esophageal squamous cell carcinoma according to the morphology of intrapapillary capillary loops. **(A)** Diagram of superficial vascular network of normal esophageal mucosa (quoted from Inoue H et al., Ann Gastroenterol 2015; 28: 41-48). **(B)** Magnified endoscopic images under NBI of different IPCL types. M1, M2 and M3 are epithelium infiltration, lamina propria infiltration and Muscularis mucosa infiltration, respectively. SM1 and SM2 refer to the lesion infiltrating the upper 1/3 of the submucosa and the middle 1/3 of the submucosa, respectively.

Based on magnified electron chromoendoscopic images of intrapapillary capillary loop (IPCL), the Japanese Esophagus Society proposed an easy-to-understand JES classification for early ESCC (22). Blood vessels found in normal or inflammatory tissues are Type A, while those found in cancer tissues are Type B. Type B is further divided into B1, B2, and B3 subtypes. Type A IPCL showed no change or a slight change, while Type B IPCL showed obvious morphological changes. B1: IPCL dilation, bending, different diameters, and inconsistent shapes, mainly involves the epithelium (M1) and lamina propria (M2); B2: abnormal IPCL that is difficult to form a ring, mainly involves mucosal muscular (M3) and the superficial submucosa (SM1); B3: highly dilated, irregular blood vessels, mainly involves the submucosa that is 200 mm (SM2) or deeper (SM2) (**Figure 1**). The AB classification of IPCL in esophageal lesions under magnification NBI endoscopy (ME-NBI) is helpful to predict the nature and infiltration depth of esophageal lesions, to achieve comprehensive assessment of the disease and to develop the best treatment strategy for patients (23). This classification is often used clinically to determine the depth of invasion of superficial ESCC.

## 3 Limitations of endoscopy for early esophageal cancer screening

Along with the development of high-definition digital technology, standard high-definition WLI endoscopes (HD-WLI) can produce high-definition image signals with resolutions up to megapixel level. Assessment of mucosal surface morphology and vascular status using endoscopic images allows for the timely diagnosis of dysplasia or early cancer, improving our ability to detect subtle esophageal mucosal lesions. However, the visual identification of dysplasia and early esophageal cancer using HD-WLI endoscopy is still a very challenging task because endoscopic diagnosis is highly subjective and requires a lot of technical learning and accumulated experience.

According to statistics, ordinary WLI endoscopy has a misdiagnosis rate of up to 40% for early esophageal squamous cell dysplasia or early ESCC (24). Iodine chromoendoscopy is recommended for ESCC screening in high-risk groups for esophageal cancer. Although the sensitivity of this method is > 90%, it only has a low specificity of about 70%, and this method is time-consuming and laborious, increases the workload of clinicians, and also brings the risk of allergic reactions (25). The compliance rate during the daily examinations is also not high. Advanced endoscopic imaging using NBI, and other image enhancement systems can detect ESCC with a high degree of sensitivity, but a randomized controlled trial showed that its specificity is only about 50%, and there is still much room for improvement of diagnostic accuracy (26).

Limitations of current methods for identifying esophageal tumors have spurred the development of several new techniques with enhanced diagnostic capabilities (11, 27–31). Some commercially available imaging techniques, such as probe-based confocal endoscopy (pCLE) have claimed to be comparable to pathological slides and have aimed to replace random biopsy imaging techniques, but a lot of specialized training is needed to interpret the results (31). Moreover, they are not accurate enough and too expensive at present, which limits their application, and currently, their clinical application is not common. Furthermore, even pathologists have poor consistency in identifying low-grade dysplasia (LGD), which also leads to missed diagnoses and disease progression.

The accuracy of endoscopy results largely depends on the professional level of the endoscopist. Studies have shown that inexperienced endoscopists (less than 5 years and 1000 cases of endoscopy) caused missed diagnoses of early esophageal cancer presenting as smaller lesions (32). The large demand for endoscopy examination in screening for early esophageal cancer increases the burden of clinical work, and thus the increase in endoscopist fatigue may affect the efficiency and accuracy of the examination. Endoscopic diagnosis assisted by artificial intelligence (AI) is an effective way to solve the problem of a sharp increase in the workload of endoscopic examinations and low inspection efficiency.

## 4 Basic concept of convolutional neural network

Due to the limitations of current methods of detecting esophageal tumors, technologies are being developed, and an increasing number of computer-aided diagnostic (CAD) techniques for assessing endoscopic images have evolved into auxiliary endoscopy tools. The auxiliary diagnosis system based on the latest artificial intelligence technology has become the focus of attention. However, for those without a background in computer science, the term “artificial intelligence” seems daunting.

The concept of artificial intelligence first refers to the ability of computers to perform tasks that may imitate human thinking, mainly through the “cognitive” function, to obtain the ability to “learn” and “solve problems”. Later, scientists came up with the term “machine learning” (ML), which means that computer systems without specific programming can acquire the ability to “learn” by using data and develop predictive mathematical models based on input data by identifying “features” (33). ML is a subset of AI that empowers machines or systems to automatically enhance their ability to make decisions by processing data. “ML models” can adapt to new situations and predict and make decisions in new situations. For example, if thousands of car and truck images are provided to the ML algorithm, the algorithm will eventually be able to classify new images as cars or trucks (34).

Deep learning (DL) is a subset of ML and an artificial neural network in ML. Multiple neural nodes in the network (similar to human neurons) are connected, and the neural nodes in the network are the characteristic faces of the given dataset identified in the data conversion layer. DL imitates the neurons of the human brain to learn data and transmit information, to learn how to classify data. For example, we once again provide the DL algorithm with images of cars and trucks. This time, it will learn to recognize the features of each type of vehicle (this is the key point of DL) to classify new vehicle images. DL’s ability to recognize features and learn without manual supervision makes it widely used in the medical field because there is sufficient visual data there that needs analysis and interpretation (35).

The key term in the AI field is “convolutional neural network” (CNN). CNN is a type of deep feedforward neural network with convolutional computation that is developed based on a depth neural network (36). It can better obtain the spatial position and shape information of images and is conducive to image classification. It is one of the most common methods modern DL algorithms use to complete its feature recognition. CNN is based on the way that neurons in the visual cortex react specifically in the presence of certain visual stimuli (36). The basic structure of CNN consists of an input layer, a convolutional layer, a pooling layer (also called a sampling layer), a full connection layer, and an output layer. The “convolution layer” is a filter hovering over the image (i.e., convolved on the image) that extracts the key features of the image. Each feature of the image extracted by these “filters” will generate an “activation map,” which essentially highlights the extraction function of the “filter.” This “activation map” is also a pooling layer, which plays the role of secondary feature extraction. Such a convolution layer is connected to a pooling layer to form a convolution unit. Since each neuron of the output feature surface in the convolution unit is locally connected with its input graph, the corresponding connection weight value is weighted and summed based on the local input to obtain the input value of the neuron (37).

**Figure 2:**
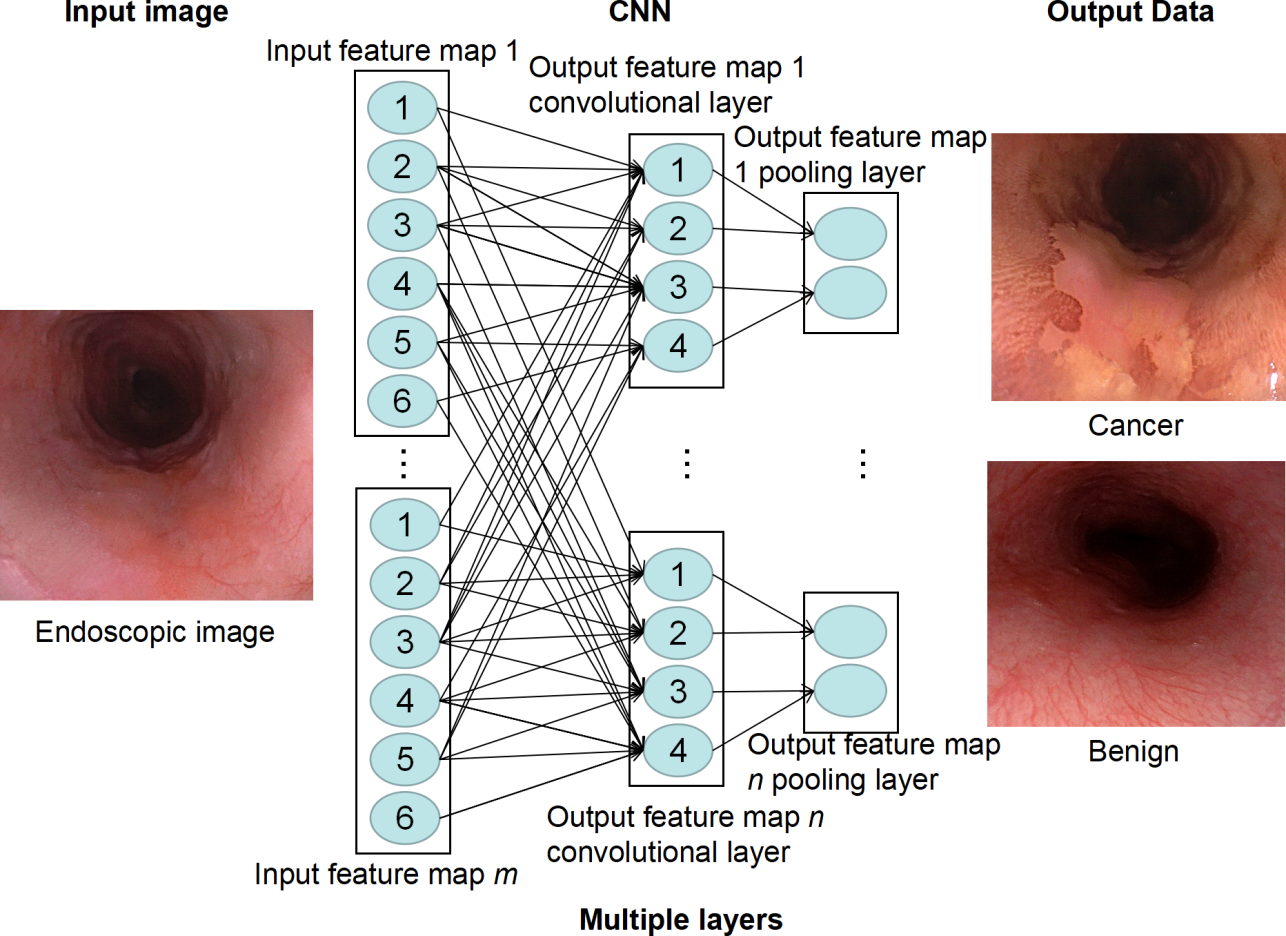
Deep learning model of convolutional neural network (CNN). CNN extracts feature of endoscopic images through multiple neural layers to predict whether there is esophageal cancer in the image.

Each subsequent layer of the CNN works on the activation map of the previous layer, resulting in the recognition of increasingly complex features as the network gets deeper and deeper. The goal of a CNN is to develop filters that hover over an image to identify features. In the CNN model, there are multi-layer image perceptrons (equivalent to artificial optic neurons), multiple neural network layers, continuous convolutional layers, and rear pooling layers. The convolution layer mainly extracts key features from the input image. After multiple image features enter the model and pass through multiple layers of CNN, the unimportant features are automatically filtered out to complete the extraction of key features in the image (37). Finally, CNN outputs the classification decision results through the full connection layer by associating specific features with each category (**Figure 2**). To effectively carry out such correlation and assign appropriate weights to the given features, CNN requires the data to be self-trained. After multiple trainings on labeled images, CNNs are capable of testing new datasets by fine-tuning their layers. The larger the number and the higher the quality of the dataset in the training phase, the more accurate the CNN is likely to be in the testing phase.

## 5 Application status of AI for the diagnosis of early ESCC

Based on HD-WLI endoscopy, electronic chromoendoscopy, chemical chromoendoscopy, magnifying endoscopy, and other methods, AI-assisted diagnosis technology realizes the functions of lesion recognition, analysis, and real-time marking of lesion areas during the process of endoscopic examination through the deep learning of the characteristics of pathological endoscopic images. At present, researchers have conducted extensive exploration of AI-assisted lesion detection, lesion extent marking, depth of invasion prediction, and real-time identification of early ESCC. As shown in **Table 1**, most of the studies at present are mainly focused on the establishment of AI algorithms based on the retrospective study of single-center data. **Table 1** summarizes all the studies investigating the development of deep learning algorithms for the diagnosis of ESCC up to date. All the ESCCs included in the studies were confirmed histologically by endoscopic submucosal dissection, surgery or biopsy.

**Table 1:** Characteristics of studies investigating the AI for early ESCC.

References	Study design	Study aim	Images mode	AI model	External validation	Patients in training set	Images for training	Patients in test set	Images for test
Everson 2019 (38)	R	Differentiation of abnormal IPCL(B1/B2/B3) from normal (A)	ME-NBI	CNN	No	17 (10 ESCCs)	7046*	17 (10 ESCCs)	7046*
Zhao 2019 (39)	R	Detection of ESCC	ME-NBI	CNN-SVM	No	219(165 ESCCs)	1383^&^	219 (165 ESCCs)	1383^&^
Nakagawa 2019 (40)	R	Determining the invasion depth Of ESCC	WLI, non-ME and ME-NBI/BLI, LCE	DCNN	Yes	804 ESCCs	14,338 (5678 ME images)	155 ESCCs	914 (509 ME images)
Cai 2019 (41)	R	Localize and identify ESCC	WLI	DNN	No	746	2428 (1332 ESCCs)	52	187
Guo 2019 (42)	R	Real-time diagnosis of ESCCs	NBI	CNN-SegNet	No	549 (191 ESCCs)	6473	60 (27 early ESCCs)	80 videos
Ohmori 2020 (43)	R	Detect and differentiate ESCC.	WLI, non-ME and ME NBI/BLI, LCE	CNN -SSMD	No	Unknown (804 ESCCs)	22,562 (11279 ME images)	135 (52 ESCCs)	727 (204 ME images)
Tokai 2020 (44)	R	Measure ESCC invasion depth	WLI and NBI	CNN-GoogLeNet	No	55	1751	291	291
Fukuda 2020 (45)	R	Diagnosing ESCC with videos	NBI or BLI	CNN-SSMD	No	2002 (1544 ESCCs)	28,333	144	144 videos
Shimamoto 2020 (46)	R	Calculate cancer invasion depth	WLI, non-ME and ME NBI/BLI, LCE	CNN -PyTorch	No	909	23,977 (17,120 NBI/BLI)	102	102 ESCC videos
Everson 2021 (47)	R	Recognition of IPCL patterns and predict invasion depth	ME- NBI	CNN-ResNet-18	No	114 (69 dysplastic)	67,740 (39,662 dysplastic , five-fold cross validation)	114 (69 dysplastic	67,740 (39,662 dysplastic , five-fold cross validation)
Tang 2021 (48)	R	Real-time to diagnose ESCC	WLI	DCNN	Yes	1,078 (337 ESCCs)	4,002	162 (58 ESCCs)	700 (207 ESCCs)
Ikenoyama 2021 (49)	R	Predict multiple LVLs	WLI and NBI	CNN- GoogLeNet and Caffe	No	595 (188 with multiple LVLs)	6634 (2736 with multiple LVLs)	72 (32 with multiple LVLs)	667 (342 with multiple LVLs)
Yang 2021 (50)	R	Automatic diagnosis of early ESCC	WLI, non-ME and ME-OE images, LCE	DCNN -Yolo V3, ResNet V2	No	5075 cases	10,988	1055 cases	2309 images, 104 videos
Uema 2021 (51)	R	Classify the microvessels of ESCCs	ME-NBI	CNN-ResNeXt-101	No	262 lesions	1777 (1134 B1, 557 B2, 86 B3)	131 lesions	747 (419 B1, 292 B2, 36 B3)
Waki 2021 (52)	R	Detect ESCC with Videos without focusing on the lesion	WLI, NBI, and BLI	DL-BiSeNet	No	1763 (1567 ESCCs)	18,797 (17,336 ESCCs)	100 (50 ESCCs)	100 videos (50 ESCCs)
Shiroma 2021 (53)	R	Detect ESCC from enoscopic videos	WLI and NBI	DCNN-SSMD	No	397 ESCCs (65 advanced cancers)	8428	72 patients	144 Videos
Wang 2021 (54)	R	Detect and differentiate histological grade of ESCC	WLI and NBI	CNN-SSD	No	Unknown (46 ESCC)	936 (162 normal )	Unknown	264 (54 normal)
Li 2021 (55)	R	Identify ESCC under NBI imaging and compare it with WLI	NBI and WLI	CNN-VGG	No	647 (235 ESCCs)	4735 (2167 ESCCs)	112 cases (42 ESCCs)	316 pairs of images (133 abnormal)
Yuan 2022 (56)	R	Detecting ESCC	WLI, non-ME and ME NBI, LCE	DCNN -YOLO v3	Yes	2291 ESCCs	45,770 (29 248 cancerous images)	119	2088 (1245 ESCCs), 142 videos (76 ESCCs)
Yuan 2022 (57)	R	Predict IPCLs subtypes of ESCC	ME-NBI	DCNN -HRNet+OCR	Yes	496 lesions	5505	176 patients	1323
Liu 2022 (58)	R	Detect and delineate margins of ESCC	WLI	DCNN-YOLACT	Yes	977	10,467 (4885 ESCCs)	312 (96 external validation)	3506 (890 external validation)
Tajiri 2022 (59)	R	Detect ESCC in simulated clinical situations	WLI, ME and non-ME NBI/BLI	BiT-M (ResNet-101×1)	No	1843 (1433 ESCCs)	29,794 (25,048 ESCCs)	147 lesions (83 ESCCs)	147 videos (83 ESCC videos)
Wang 2022 (60)	R	Diagnosis of ESCC	WLI, NBI and LCE	DL-YOLOv5l	No	1025	11, 547	101	1462
Yuan 2023 (61)	R and P	Detect and delineate the extent of ESCC	NBI	DCNN-YOLACT	Yes	899 (802 ESCCs)	7530 (4512 ESCCs)	414 cases (311 ESCCs)	2517 (1488 ESCCs) and 140 videos (70 ESCCs)

R, Retrospective; P, Prospective; ESCC, Esophageal squamous cell carcinoma; IPCL, Intrapapillary capillary loop; WLI, White light imaging; NBI: Narrow band imaging; LCE, Lugol chromoendoscopy; ME: Magnifying endoscopy; LVL, Lugol-voiding lesions; CNN, Convolutional neural network; DCNN, Deep convolutional neural network; DNN, Deep neural network; DL, Deep learning; OE optical enhancement; * The images were five-fold cross-validation; ^&^ The images were three-fold cross-validation.

### 5.1 Application of AI-assisted WLI in early ESCC detection

WLI has become the first choice for the diagnosis of early ESCC. AI can help inexperienced endoscopists intelligently analyze medical images to better detect and classify lesions. In 2019, Cai et al. used CNN to develop a computer-aided detection (CAD) system for identifying early ESCC based on conventional WLI endoscopy. The system was trained on 2,428 (1,332 abnormal and 1,096 normal) esophageal endoscopic images of 746 patients obtained from 2 centers. The validation dataset contained 187 images from 52 patients. The results showed that the diagnostic sensitivity, specificity, and accuracy of the CAD system were 97.8%, 85.4%, and 91.4%, respectively. The area under the receiver operating characteristic curve (AUC) was >96%. The diagnostic accuracy of senior endoscopists was 88.8%, while that of junior endoscopists was 77.2%. The average diagnostic ability of endoscopists improved after referring to CAD results, especially in terms of sensitivity (74.2% *vs*. 89.2%), accuracy (81.7% *vs*. 91.1%), and NPV (79.3% *vs*. 90.4%) (41). This suggests that the CNN-CAD system has high accuracy and sensitivity for early ESCC screening and can help endoscopists detect lesions that were previously overlooked under WLI.

Another study from China in 2021 reported on the diagnosis of early-stage ESCC using WLI based on a real-time deep convolutional neural network (DCNN) system. A total of 4,002 images from 1,078 patients were used to train and cross-validate the DCNN model. The diagnostic performance of the model was validated with a total of 1,033 images from internal and external datasets of 243 patients. It was found that the DCNN model performed excellently in diagnosing early ESCC, with a sensitivity of 0.979, a specificity of 0.886, and an AUC of 0.954. The diagnostic accuracy of endoscopists was significantly improved after referring to the prediction results of the DCNN model (48). The research results in 2021 and 2022 also suggest that the AI model under WLI can accurately identify early ESCC (50, 58). Since WLI is the most widely used examination method in daily practice, AI-assisted WLI for early diagnosis of ESCC has great clinical significance and is a potential assistant for endoscopists, especially for young endoscopists.

### 5.2 The value of AI-assisted non-magnified electronic chromoendoscopy for the diagnosis of early ESCC

The non-magnifying narrow-band imaging (NM-NBI) endoscopy system improves the visualization of microvessels and mucosal patterns in the digestive tract. The NM-NBI has been used for routine screening of ESCC, which has higher accuracy and specificity than ordinary WLI. Li et al. constructed an AI-aided diagnosis system based on NBI images and compared the value of their previously established AI-aided diagnosis system based on WLI images for the diagnosis of early esophageal cancer. The training dataset contains 2167 abnormal NBI images of 235 early-stage ESCC patients and 2,568 NBI images of 412 normal patients from three institutions. Then, they collected 316 pairs of images (133 pairs of abnormal and 183 pairs of normal) as a test dataset, each pair of images including WLI and NBI images at the same position and angle. It was found that the AUC of CAD-NBI was 0.9761. The diagnostic sensitivity, specificity, accuracy, positive predictive value, and negative predictive value of the CAD-NBI system were 91.0%, 96.7%, 94.3%, 95.3%, and 93.6%, respectively, while the CAD-WLI was 98.5%, 83.1%, 89.5%, 80.8%, and 98.7%. CAD-NBI showed higher accuracy and specificity than CAD-WLI, while CAD-WLI was more sensitive than CAD-NBI. By using CAD-WLI and CAD-NBI together, endoscopists can improve their diagnostic efficiency to the highest accuracy, sensitivity, and specificity of 94.9%, 92.4%, and 96.7%, respectively (55).

Wang et al. constructed a single-shot multi-box detector (SSD) system using a neural convolution algorithm and tested its accuracy in the diagnosis of esophageal neoplasms and its performance in differentiating histological grades. A total of 936 endoscopic images were used to train the system, including 498 WLI images and 438 NBI images. Esophageal neoplasms were divided into three categories based on pathological diagnosis: low-grade squamous dysplasia, high-grade squamous dysplasia, and squamous cell carcinoma. The AI system analyzed 264 test images within 10 seconds, including 112 WLI images and 152 NBI images. SSD accurately diagnosed 202 of 210 esophageal neoplasm images and 38 of 54 normal esophageal images. The diagnostic sensitivity, specificity, positive predictive value (PPV), negative predictive value (NPV), and accuracy were 96.2%, 70.4%, 92.7%, 82.6%, and 90.9%, respectively. Comparing the diagnostic performance between WLI and NBI, SSD-WLI images showed higher specificity and PPV, while NBI images showed higher sensitivity and NPV. Overall, SSD diagnostic WLI images and NBI images had similar accuracy. The overall accuracy of SSD in diagnosing esophageal neoplasms of different histological grades was 92%, and the accuracy of SSD in diagnosing NBI images was 95%, which was higher than that of WLI images (89%). SSD showed good sensitivity to ESCC, and the sensitivity of WLI and NBI images to esophageal SCC was 97% and 100%, respectively (54).

Ohmori et al. found that an AI system can diagnose 52 (100%) of 52 SCC cases as cancer, 33 (100%) of 33 normal mucosa cases as normal mucosa, and 19 (38%) of 50 non-cancerous esophageal lesions as non-cancerous lesions through NBI/BLI images. On average, experienced endoscopists diagnosed 48 (92%) of 52 SCCs as cancer, 31 (94%) of 33 normal mucosae, and 26 (52%) of 50 non-cancerous lesions as non-cancerous lesions. The AI system accurately diagnosed SCC and normal esophagus in all samples without producing false negative or false positive results. The sensitivity of AI for SCC diagnosis was better than that of experienced endoscopists, but its specificity for non-cancerous lesions was lower than that of experienced endoscopists. The AUC of the validation dataset using NBI/BLI is 93% (43).

Yuan et al. have recently reported an AI system for detecting and delineating ESCC under NBI. The system was trained with 7530 images from 899 lesions, of which 4512 were ESCC images from 802 lesions. The retrospectively collected 1376 images from 130 lesions containing 804 ESCC images and 1141 image from 111 lesions containing 684 ESCC images were used as internal test dataset and external test dataset, respectively. The accuracy of the system in detecting ESCC in internal and external tests was 92.4% and 89.9%, respectively, while the accuracy of the system in delineating extents in internal and external tests was 88.9% and 87.0%, respectively. They also prospectively collect 140 videos including 70 cancerous lesions to perform clinical evaluation, the system also showed satisfactory performance, with an accuracy of 91.4% in detecting lesions and an accuracy of 85.9% in delineating extents (61). It can be seen from the above research that endoscopists can improve diagnostic efficiency by using AI-assisted NBI images. It helps to avoid missed diagnosis and excessive biopsy, which may help endoscopists, especially those with less experience, screen for early ESCC more effectively.

### 5.3 The role of AI-assisted magnifying electronic chromoendoscopy in the diagnosis of ESCC

Magnifying electronic chromoendoscopy (ME-NBI/BLI) improves the visualization of subtle changes in intramucosal capillary loops (IPCL), which play a key role in the diagnosis of early ESCC (62). However, the accuracy of IPCL classification depends on the operator’s experience and requires an objective and accurate method to evaluate.

Everson et al. reported that a total of 7046 ME-NBI images sampled at 30 fps from videos of 17 patients (10 ESCCs, 7 normal) with 5-fold cross-cycle validation were used to train CNN. Three endoscopic experts classified the IPCL pattern of the images into normal (type A) and abnormal (types B1–3) using the Japanese Society of Endoscopy criteria as the gold standard. All imaging was verified histologically. The results showed that the accuracy of the CNN in diagnosing normal and abnormal IPCL patterns was 93.7% (86.2% to 98.3%), the sensitivity was 89.3% (78.1% to 100%), and the specificity was 98% (92% to 99.7%). It shows that CNN can accurately classify IPCL patterns as normal or abnormal. And the prediction time of the CNN diagnosis is between 26.17 ms and 37.48 ms (38). The extremely fast diagnostic speed of the system indicates that it can be used for real-time clinical decision-making *in vivo* as a support tool to guide endoscopists to evaluate ESCC.

Zhao et al. conducted a study on the diagnostic value of AI-assisted ME-NBI images for early esophageal cancer in a total of 219 patients, including 30 inflammatory lesions, 24 low-grade intraepithelial neoplasia, and 165 early esophageal cancers. Endoscopy experts combined pathological results to determine the gold standard diagnosis of IPCL. The endoscopic gold standard for IPCL diagnosis in 219 patients was 31 type A, 143 type B1, and 45 type B2. The dataset was validated using a 3-fold cross-validation in the study. Among the 1383 lesion images in the study, the average accuracy of IPCL classification by advanced, intermediate, and junior group endoscopists was 92.0%, 82.0%, and 73.3%, respectively. The model achieved an average diagnostic accuracy of 93.0% at the image level. The diagnostic accuracy rate of inflammatory lesions in the model (92.5%) was higher than that of intermediate-level (88.1%) and primary-level (86.3%) endoscopists. The diagnostic accuracy of the model for malignant lesions (B1, 87.6%; B2, 93.9%) was significantly higher than that of the intermediate (B1, 79.1%; B2, 90.0%) and junior (B1, 69.2%; B2, 79.3%) endoscopist groups (39).

Ohmori et al. developed a computer image analysis system to detect and differentiate ESCC. They tested 204 ME-NBI/BLI images from 135 patients. The diagnostic sensitivity, specificity, and accuracy of ME-NBI/BLI assisted by a computer image analysis system were 98%, 56%, and 77%, respectively, while the diagnostic sensitivity, specificity, and accuracy of 15 experienced endoscopy doctors were 83%, 70%, and 76%, respectively (43). There is no significant difference in diagnostic performance between AI systems and experienced endoscopists.

Everson MA used a dataset containing 67,742 high-quality ME-NBI images to train CNN for IPCL classification. These images were extracted at 30 frames per second from examination videos of 114 patients (45 with normal mucosa and 69 with dysplasia). Through the method of five-fold cross training and verification, a total of 28,078 normal images and 39,662 dysplasia images were collected, with an average of 593 frames per patient. This study also evaluated the IPCL classification results of 158 representative images by 5 Asian and 4 European endoscopy experts. The results showed that the F1 scores (the measurement accuracy of binary classification) of European experts and Asian experts were 97.0% and 98%, respectively. The sensitivity and accuracy of European and Asian endoscopic specialists were 97% and 98% and 96.9% and 97.1%, respectively. The average F1 score of CNN was 94%, and the average diagnostic sensitivity and accuracy were 93.7% and 91.7%, respectively (47). It is shown that the CNN achieves diagnostic performance comparable to that of an endoscopic expert panel.

### 5.4 The role of AI in the diagnosis of ESCC invasion depth

The Paris classification based on white-light endoscopic morphology has a certain value in judging the depth of tumor invasion and guiding the choice of treatment, but it lacks accuracy in judging the depth of invasion in early esophageal cancer. During the progression of ESCC, the initial slender annular structure of IPCLs becomes more tortuous and dilated and then forms a linearly dilated vascular structure. In the deeper submucosal infiltration stage, these structures disappear and are replaced by neovascularization consisting of tortuous, dilated, and acyclic capillaries. The morphological changes of IPCL are related to the depth of tumor invasion, which is the main factor in determining endoscopic treatment. However, the current diagnosis of invasion depth is subjective and has obvious individual differences (63). Therefore, a computer-aided diagnosis system that can objectively classify and diagnose is needed.

Nakagawa et al. developed an AI-assisted diagnostic system based on SSD to evaluate the invasion depth of superficial ESCC. The training dataset consists of 8,660 non-magnified and 5,678 magnified endoscopic images collected from 804 patients with superficial ESCC with a pathologically confirmed depth of cancer invasion. 405 non-magnified endoscopic images and 509 magnified endoscopic images were selected from 155 patients for validation. The results showed that the system had a sensitivity of 90.1%, a specificity of 95.8%, a PPV of 99.2%, an NPV of 63.9%, and an accuracy of 91.0% for correctly distinguishing intramucosal/slightly invasive carcinoma (SM1) from submucosal deeply invasive (SM2/3) carcinoma, whereas 16 experienced endoscopists using the same validation set correctly differentiated intramucosal/micro-invasive carcinoma (SM1) from submucosal deeply invasive (SM2/3) carcinoma with 89.8% sensitivity, 88.3% specificity, 97.9% PPV, 65.5% NPV, and 89.6% accuracy (40). This shows that this AI system has a good performance in diagnosing the invasion depth of superficial ESCC, and its performance is comparable to that of experienced endoscopists.

Uema et al. retrospectively collected a total of 393 ME-NBI images of superficial ESCC and performed a diagnostic study on the classification of IPCL morphology by a CNN-based AI diagnostic system. All images were evaluated by three experts based on the Japan Esophagus Society classification of IPCL and classified into three categories: B1, B2, and B3. 1,777 images were used as a training dataset, and the remaining 747 images were used as a validation dataset. In the training dataset, 1,134 images were labeled as type B1, 557 as type B2, and 86 as type B3. Correspondingly, in the validation dataset, 419 images were labeled as type B1, 292 as type B2, and 36 as type B3. The results showed that the accuracy of the CAD system for microvessel classification was 84.2%, which was higher than that of eight endoscopists (77.8%, P 0.001). The AUCs for diagnosing B1, B2, and B3 vessel types were 0.969, 0.948, and 0.973, respectively (51). The CAD system shows superior performance in the classification of microvessels in superficial ESCC.

Yuan et al. recently reported an AI-assisted diagnosis system based on a DCNN algorithm named HRNet+OCR to diagnose the microvascular morphology of lesions. A total of 7,094 ME-NBI images from 685 patients were used to train and validate the AI system. The comprehensive accuracy of the AI diagnostic system for diagnosing IPCL subtypes in the internal and external validation datasets was 91.3% and 89.8%, respectively. It is superior to the senior endoscope in diagnosing IPCL subtypes and predicting the depth of lesion invasion. With the assistance of the AI system, junior endoscopists significantly increased the comprehensive accuracy of the diagnosis of IPCL subtypes from 78.2% to 84.7% and the comprehensive accuracy of the depth of invasion from 67.9% to 74.4%. Although there was no significant difference, the diagnostic accuracy of senior endoscopes was improved with the help of the AI system (57). From the above results, it can be seen that the AI system can improve the diagnostic ability of endoscopists for the IPCL classification of precancerous lesions and superficial ESCC.

### 5.5 The role of AI in the real-time diagnosis of ESCC

AI studies using static images may have some bias because these studies typically select the best-imaged regions for analysis. The performance of the AI diagnosis system on video images can be evaluated in real-time and was found to be closer to the actual situation in clinical application.

Guo et al. developed a CAD system to help with the real-time automatic diagnosis of precancerous lesions and early ESCCs. The system used 2,770 NBI images from 191 cases of early ESCCs or precancerous lesions and 3703 NBI images from 358 cases of non-cancerous lesions, including esophageal varices, ectopic gastric mucosa, esophagitis, and normal esophagus, as training data sets. Twenty-seven cases of precancerous lesions or early ESCC confirmed by pathology, including 27 non-magnified videos and 20 magnified videos, and 33 cases of normal esophagus, including 30 non-magnified videos and 3 magnified videos, totaling 80 video fragments in total, were randomly selected as the test dataset. The results showed that the overall sensitivity and specificity of CAD per frame were 91.5% and 99.9%, respectively. In precancerous lesions or early esophageal cancer, the diagnostic sensitivity of non-magnified video and magnified video per frame was 60.8% and 96.1%, respectively. The diagnostic sensitivity of a single lesion in non-magnified and magnified video clips was 100%. In normal esophageal cases, the diagnostic specificity of each case was 90.9% (42). This demonstrates the high sensitivity and specificity of the deep learning model for video dataset diagnosis.

Fukuda et al. reported on the results of using AI for the real-time diagnosis of early ESCC. They used 23,746 images from 1,544 cases of pathologically confirmed superficial ESCCs and 4,587 images from 458 cases of non-cancerous and normal tissues to build an artificial intelligence (AI) system. The 5- to 9-second video clips of 144 patients taken with NBI or BLI were used as the validation dataset. These video images were diagnosed by the AI system and 13 certified endoscopists, respectively. The results showed that the sensitivity, specificity, and accuracy of the AI system and experts in diagnosing ESCC from non-magnified NBI video were 91%, 51%, and 63%, and 79%, 72%, and 75%, respectively. The sensitivity, specificity, and accuracy of the AI system and experts in diagnosing early SCC with magnified NBI video were 86%, 89%, 88%, and 74%, 76%, and 75%, respectively (45). It shows that the sensitivity of the AI system is significantly higher than that of experts, but its specificity is significantly lower than that of experts.

Tajiri et al. reported the latest results of real-time diagnosis of early ESCC. They used 25,048 images from 1,433 cases of superficial ESCC and 4,746 images from 410 cases of the non-cancerous esophagus to build the AI system. The validation dataset is NBI videos of suspected superficial ESCC. These videos correspond to sequential videos of routine diagnostic procedures, including the detection of lesions by non-magnified images, approaching lesions, and observation of microvascular patterns by magnified images. The AI system also used a zoomed-in, still image captured from each video for diagnosis. The 147 videos in the validation dataset included 83 cases of superficial ESCC and 64 cases of non-ESCC lesions. The accuracy, sensitivity, and specificity of the AI system for ESCC classification were 80.9% [95% CI 73.6–87.0], 85.5% [76.1–92.3], and 75.0% [62.6–85.0], respectively, while the accuracy, sensitivity, and specificity of the endoscopy were 69.2% [66.4–72.1], 67.5% [61.4–73.6], and 71.5% [61.9–81.0], respectively (59). It was suggested that the AI system showed higher accuracy than endoscopists in diagnosing ESCC and non-ESCC. It can provide valuable diagnostic support to endoscopists.

Shimamoto et al. developed an AI system for real-time diagnosis of ESCC invasion depth. A total of more than 20,000 white light and magnified and non-magnified NBI/BLI images of ESCC with pathologically determined infiltration depth were used as training datasets. 102 ESCC video images serve as an independent validation dataset. Each case included two types of videos: a 4–12 second magnified NBI/BLI video and a non-magnified WLE video. The diagnostic accuracy, sensitivity, and specificity of the AI system and the endoscopist on non-magnified videos were 87%, 50%, and 99%, and 85%, 45%, and 97%, respectively. The accuracy, sensitivity, and specificity of the AI system and the endoscopist on magnifying videos were 89%, 71%, 95%, and 84%, 42%, and 97%, respectively (53). It is suggested that the two types of video AI systems have better diagnostic accuracy, sensitivity, and specificity than those of the expert group. The AI model can effectively assist in evaluating the depth of the ESCC invasion in real-time.

## 6 Conclusions and perspectives

At present, AI diagnostic systems developed based on different algorithms and image modalities (such as static images or dynamic video, WLI/NBI, and magnified/non-magnified images) have demonstrated equivalent or superior diagnostic accuracy, sensitivity, and specificity compared to endoscopic experts. However, the majority of studies have focused on establishing, training, and validating AI algorithms utilizing retrospective data from a single center. The quality of the learning resources within the training dataset significantly impacts the diagnostic performance of the model. The disparity between the training dataset and the actual endoscopic working environment may limit its clinical applicability.

Currently, ESCC endoscopic screening relies primarily on WLI and NBI endoscopy in common clinical practice. The establishment of an AI system for real-time detection and delineation of the ESCC based on WLI and NBI video is most suitable for clinical use. Yuan et al. have reported an AI system established by the WLI and NBI imaging modes can be directly connected to endoscopic monitor, and the AI system can detect and delineate very small flat lesions in real-time fashion (64), which provides a model of the clinical application of an AI in endoscopic diagnosis of ESCC in the future. However, there is still a need to integrate diverse datasets from multi-center, multi-equipment, and multiple imaging modalities to optimize and iterate a more robust and compelling AI system. Additionally, external multicenter validation, especially prospective multicenter double-blind randomized controlled trials, is required to confirm the accuracy of the results before AI can be applied to clinical practice.

Although there are still many obstacles before the large-scale application of AI in ESCC diagnosis, the application of AI during early ESCC detection has shown promising prospects. The increasing number of people participating in endoscopic screening for ESCC leads to an increase in the workload of endoscopists. An AI algorithm can achieve rapid and accurate diagnosis in seconds or minutes, which is conducive to reducing the workload of endoscopy doctors. Shortly, advanced AI systems will be compulsorily incorporated into the composition of endoscopic equipment for daily usage to improve clinical outcomes. More and more patients and physicians will benefit from the progress of endoscopic AI auxiliary diagnosis systems.

## Author contributions

YP and LH: writing—original draft. WC: writing—review and editing. YY: conceptualization, writing—review and editing, figure drawing, and funding acquisition. All authors have read and approved the submitted version of the manuscript. All authors contributed to the article.
